# Persisting with prevention: The importance of adherence for HIV prevention

**DOI:** 10.1186/1742-7622-5-8

**Published:** 2008-07-11

**Authors:** Helen A Weiss, Judith N Wasserheit, Ruanne V Barnabas, Richard J Hayes, Laith J Abu-Raddad

**Affiliations:** 1 Medical Research Council Tropical Epidemiology Group, Department of Epidemiology and Population Health, London School of Hygiene & Tropical Medicine, London, UK; 2 University of Washington School of Medicine, Seattle, Washington, USA; 3 HIV Vaccine Trials Network, Fred Hutchinson Cancer Research Center, Seattle, Washington, USA; 4 Program in Biostatistics and Biomathematics, Vaccine and Infectious Disease Institute, Fred Hutchinson Cancer Research Center, Seattle, Washington, USA

## Abstract

**Background:**

Only four out of 31 completed randomized controlled trials (RCTs) of HIV prevention strategies against sexual transmission have shown significant efficacy. Poor adherence may have contributed to the lack of effect in some of these trials. In this paper we explore the impact of various levels of adherence on measured efficacy within an RCT.

**Analysis:**

We used simple quantitative methods to illustrate the impact of various levels of adherence on measured efficacy by assuming a uniform population in terms of sexual behavior and the binomial model for the transmission probability per partnership.

At 100% adherence the measured efficacy within an RCT is a reasonable approximation of the true biological efficacy. However, as adherence levels fall, the efficacy measured within a trial substantially under-estimates the true biological efficacy. For example, at 60% adherence, the measured efficacy can be less than half of the true biological efficacy.

**Conclusion:**

Poor adherence during a trial can substantially reduce the power to detect an effect, and improved methods of achieving and maintaining high adherence within trials are needed. There are currently 12 ongoing HIV prevention trials, all but one of which require ongoing user-adherence. Attention must be given to methods of maximizing adherence when piloting and designing RCTs and HIV prevention programmes.

## Background

Recent randomized controlled trials (RCT) of herpes suppressive therapy [1,2], female diaphragms and gel in addition to male condoms (the Methods for Improving Reproductive Health in Africa, or MIRA trial) [3], and an adenovirus-vectored HIV vaccine [4] have failed to show an impact on HIV acquisition. These disappointing findings contribute to a total of 31 completed RCTs with HIV incidence as a primary outcome for sexual transmission (Table 1), of which only four have shown a statistically significant reduction in new HIV infections [5-8].

**Table 1:** Randomised controlled trials of HIV prevention with HIV incidence as an outcome for sexual transmission

**Intervention**	**Individual or cluster randomization**	**RCTs completed or stopped**	**RCTs showing efficacy**	**RCTs ongoing**
	Individual	2	0	0
		[3,24]		
Behavior change (abstinence/delay, sex partner reduction, counseling and testing, condoms, diaphragms, microfinance)				
	Cluster	5	0	2
		[15,25-28]		[29,30]

Male Circumcision	Individual	4 ^1^	3	0
		[5-7,31]	[5-7]	

Microbicides	Individual	9	0	3
		[32-40]		[41-43]

Oral pre-exposure prophylaxis (PrEP)	Individual	1	0	4
		[35]		[44-47]

HIV Treatment	Individual	0	0	1
				[48]

	Individual	3	0	1
		[1,2,14]		[49]
STI Treatment				
	Cluster	4	1	0
		[8,15,16,50]	[8]	

HIV Vaccines	Individual	4	0	1
		[4,51-53]		[54]

**All Interventions**		**31**^2^	**4**	**12**

^1 ^The trial which did not show efficacy was of the impact of male circumcision on female HIV acquisition

^2 ^Total = 31 trials because study [15] is shown twice, under behavior change and STI treatment

Multiple factors are likely to be responsible for the failure of the trials to see a protective effect, including interventions which are truly non-efficacious, trials which were under-powered to detect an effect, and poor adherence to the intervention under study. It is striking that the only intervention for which multiple trials have shown efficacy in preventing HIV is male circumcision [5-7], a non-reversible surgical procedure for which post-intervention 'adherence' is 100%. In contrast, some of the recent RCTs of other interventions have suggested that poor adherence may have contributed to the lack of effect [1,3].

In this paper, we use simple quantitative methods to explore the impact of various levels of adherence on observed efficacy in randomized controlled trials, and discuss the implications for designing future HIV intervention trials.

## Analysis

### Methods

The impact of adherence in an RCT for an intervention with different levels of efficacy was calculated by linking the risk per sexual exposure to the cumulative risk calculated in longitudinal trials using simplifying assumptions [9]. We assume a uniform population in terms of sexual behavior and the binomial model for the transmission probability per partnership [10]. These simplifying assumptions give illustrative results of the effect of varying adherence within the population on measured efficacy within a RCT.

In the control arm, the probability of transmission per partnership during the trial is

*z*_Control _= 1 - (1 - *p*)^n*τ^

and that for the intervention arm is given by

$$z_{\text{Intervention}}=1-{\left(1-p\right)}^{n*\tau *\left(1-f_{\text{Adh}}\right)}{\left(1-\left(1-Eff_{\text{Int}}\right)*p\right)}^{n*\tau *f_{\text{Adh}}}$$

Here, *p *= 0.0015 is the average HIV transmission probability per coital act over all HIV stages for vaginal intercourse [11], *p *= 0.0082 for receptive anal intercourse [12], *n *= 10 is the frequency of coital acts per month [11], and *τ *is the average duration of the trial follow-up (here assumed to be 18 months). The term E*ff*_Int _is the biological efficacy of the intervention under study in reducing HIV transmission probability per coital act, and *f*_Adh _is the mean adherence level achieved in the trial (i.e. fraction of coital acts protected by the intervention).

Then the risk ratio (*RR*) is given by

$$RR=\frac{z_{\text{Intervention}}}{z_{\text{Control}}}$$

and E*ff*_Measured _= 1 - *RR *is interpreted as the "measured efficacy" of the intervention during the trial.

## Results

**Figure 1:**
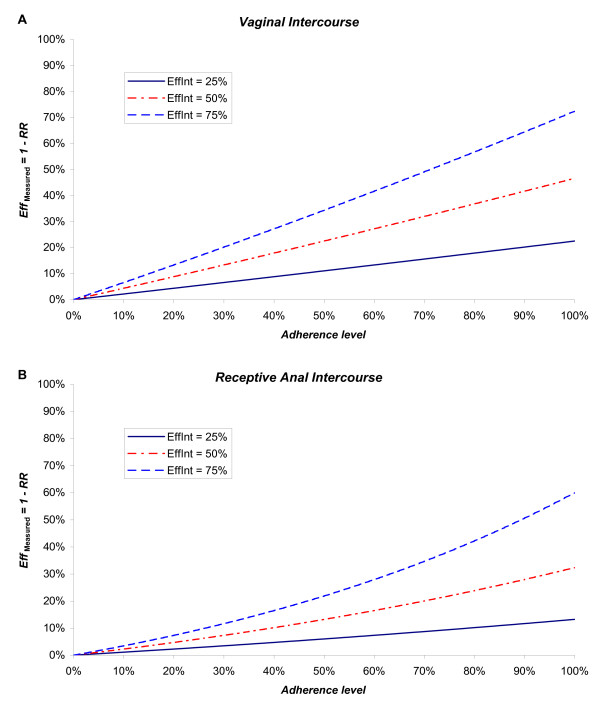
**Impact of partial adherence in randomized controlled trials. **The measured efficacy (E*ff*_Measured _= 1 - *RR*) of an HIV prevention intervention as a function of mean adherence level in a trial with actual biological efficacy per sexual act of E*ff*_Int _= 25%, 50% or 75% respectively. Panel **A **shows the results assuming vaginal intercourse as the mode of transmission and panel **B **shows the results assuming receptive anal intercourse as the mode of transmission.

Figure 1 shows the measured efficacy within the trial (E*ff*_Measured _= 1 - *RR*) as a function of mean adherence level for interventions with true biological efficacy E*ff*_Int _of 25%, 50% and 75% respectively. At 100% adherence and vaginal intercourse as the mode of transmission (Figure 1A), the measured efficacy is close to the true biological efficacy (E*ff*_Measured _of 23%, 47% and 72% respectively compared to E*ff*_Int _of 25%, 50% and 75%). However, at lower adherence levels, the measured efficacy within the trial increasingly underestimates the true biological efficacy of the intervention. For example, when adherence is 80%, the measured efficacy is considerably lower than the true efficacy (E*ff*_Measured _of 18%, 37% and 57% respectively compared to E*ff*_Int _of 25%, 50% and 75%). At adherence of 60%, the measured efficacy is just over half of the true efficacy. The absolute impact of adherence is stronger for interventions with relatively greater true biological efficacy. An extension of this model using variable adherence gave similar results (results not shown).

Similar results also hold for an intervention targeting unprotected receptive anal intercourse (Figure 1B). The measured efficacy here further underestimates the true biological efficacy even at full adherence. The measured efficacy is 13%, 32% and 60% respectively at 100% adherence compared to E*ff*_Int _of 25%, 50% and 75%. At 80% adherence, the measured efficacy is 10%, 24% and 42%; and at 60% adherence, it is 7%, 16% and 28% respectively.

## Discussion

The mean level of adherence achieved during an RCT affects the measured efficacy of the intervention, and as adherence falls, the measured efficacy will increasingly under-estimate the true biological efficacy. The main implications of this finding are that trials need to be powered to detect this smaller measured efficacy rather than the true biological efficacy, and that methods to maximize adherence within trials are urgently needed.

Many HIV prevention strategies rely on good levels of adherence. To our knowledge, 31 RCTs have reported results, of which 7 were behavioural interventions and 24 were primarily biomedical (Table 1). The behavioural interventions include voluntary counseling and testing, and educational interventions to promote abstinence, reduce number of sexual partners, and increase use of male condoms and female diaphragms. All of these rely on ongoing user-adherence. None of the behavioural RCTs found a significantly reduced risk of HIV acquisition. This may be partly due to truly ineffective interventions, low power (due for example, to under-estimates of HIV incidence, higher than expected loss to follow-up, or effective interventions in the control arm), and difficulty in achieving sustained, consistent behaviour change i.e. poor adherence.

Biomedical interventions against HIV acquisition include male circumcision, vaginal microbicides, oral pre-exposure prophylaxis (PrEP), STI treatment and HIV vaccines. The only one of these for which there has been consistent efficacy in multiple trials is male circumcision in heterosexual men. These trial results were striking both in the magnitude of effect (summary efficacy 58%, 95% CI 43%–69%) which led to all 3 trials being halted early, and also in the consistency of the results across trials and with a previous meta-analysis of observational studies [13]. One plausible reason for the strong, consistent impact of male circumcision on HIV acquisition is the in-built 100% 'adherence' of foreskin removal. Other biomedical interventions, such as treatment of bacterial STDs, also have the potential for substantial efficacy based on observational studies but only one of these RCTs afforded significant protection against HIV acquisition [8,14-16]. Reasons for the inconsistent results of the bacterial STD treatment trials have been widely discussed [17-20] and include poor adherence to the intervention as well as other factors such as the stage of the epidemic and prevalence of curable STDs in the trial population.

The importance of adherence is also suggested by the MIRA trial, and one of the HSV-2 suppressive therapy trials [1,3]. In the MIRA trial, participants in the intervention arm were asked to insert a diaphragm and use a lubricant gel before each coital act. In the HSV suppressive therapy trials, women were asked to take two tablets of acyclovir daily for up to 30 months. However, it is not clear whether suboptimal adherence was one of the factors contributing to the lack of efficacy. The median estimated adherence in the two trials was 90–94%, but was difficult to verify due to missing visits and reliance on self-report. It is possible that adherence was not the main problem in these trials, but rather that the drug or dosage used was insufficient to switch off the frequent subclinical HSV reactivations. In the Mwanza trial [1], there was little impact on detection of genital HSV, again suggesting suboptimal adherence overall, although the trial was not powered to measure an impact on this endpoint. In the MIRA trial [3], there was some evidence of impact among those women reporting high levels of adherence, and suboptimal adherence is suggested by the annual incidence of first pregnancy being similar in both intervention and control groups (13%), and consistent with the reported rates of pregnancy in these populations (13.7% in Zimbabwe in 2005–2006 [21] and 10.0% in South Africa in 1998 [22]). Moreover, the similar HIV incidence rates in both arms for each site, and across each pre-defined baseline subgroup in the trial may suggest that poor adherence was common in different sites.

Methods of achieving and maintaining high adherence both within trial and general populations must be a research priority. To our knowledge, there are currently 12 ongoing HIV prevention trials, examining a range of interventions including community-based HIV voluntary counseling and testing, vaginal microbicides, oral PrEP, herpes therapy, and an HIV vaccine (Table 1). All of these interventions, except an HIV vaccine, require ongoing user-adherence. Consistent use of pharmaceutical interventions such as, oral PrEP or vaginal microbicides may be easier to achieve than maintenance of behavioural interventions, as suggested by experience with anti-retroviral therapy, for which adherence in sub-Saharan African populations is high compared with that for condoms [23]. However, adherence to these preventive interventions is likely to be harder to maintain in uninfected individuals than adherence to therapeutic interventions, and adherence at population-level will be lower than within carefully monitored trials.

The importance of adherence in prevention trials has several implications for the design and analysis of such trials. Firstly, the anticipated levels of adherence must be taken into account when designing a trial, as sub-optimal adherence can dramatically reduce the power of the trial. Pilot studies may be most useful in estimating realistic levels of adherence, although it might be difficult to determine in advance what level of adherence would be necessary to achieve a meaningful impact. Secondly, every effort must be made to measure adherence accurately in trials and validation of self-reported adherence with biomarkers must become a priority. Thirdly, studies should ideally have adequate power to conduct sub-group analyses by adherence level, to detect whether there is an intervention effect among those with highest adherence in the absence of a significant impact overall. However, to avoid biases, these sub-group analyses would be best undertaken when the controls receive a placebo, and in practice it is unlikely that studies could be powered for such sub-group analyses. Fourthly, simulation of trial outcome using mathematical modeling at various levels of adherence can be valuable in assessing the feasibility of the trials to answer the intended research questions. Finally, when interpreting trial results, it is important to consider that a null finding does not necessarily indicate an ineffective intervention, but may reflect poor adherence to the intervention. Related to the issue of adherence within a trial is adherence during roll-out of a prevention strategy, which is likely to be lower than in controlled trials. Improved measurement of adherence within trials will help programs estimate the effectiveness of a prevention strategy during roll-out.

## Conclusion

Poor adherence during a trial can substantially reduce the power to detect an effect, and improved methods of achieving and maintaining high adherence within trials are needed. There are currently 12 ongoing HIV prevention trials, all but one of which require ongoing user-adherence. Maintaining good adherence to HIV prevention strategies will continue to be pivotal in their success. When designing and piloting both RCTs and HIV prevention programs, every effort should be made to maximize adherence.

## Competing interests

The authors declare that they have no competing interests.

## Authors' contributions

HW conceived the idea, wrote the first draft of the manuscript and led the revisions. RB compiled the list of HIV prevention trials and LAR conducted the modeling and drafted the results section. All authors commented on drafts of the manuscript and approved the final version.
